# Cambium Reactivation Is Closely Related to the Cell-Cycle Gene Configuration in *Larix kaempferi*

**DOI:** 10.3390/ijms25073578

**Published:** 2024-03-22

**Authors:** Dong-Xia Cheng, Xin-Hao Wang, Cong-Li Wang, Xiang-Yi Li, Zha-Long Ye, Wan-Feng Li

**Affiliations:** State Key Laboratory of Tree Genetics and Breeding, Key Laboratory of Tree Breeding and Cultivation of National Forestry and Grassland Administration, Research Institute of Forestry, Chinese Academy of Forestry, Beijing 100091, China; cdx@caf.ac.cn (D.-X.C.); wxh@caf.ac.cn (X.-H.W.); wangcongli@caf.ac.cn (C.-L.W.); lixiangyi@caf.ac.cn (X.-Y.L.); kemiye@caf.ac.cn (Z.-L.Y.)

**Keywords:** larch, CDKs, cyclins, cell cycle, bud burst, age, phenology

## Abstract

Dormancy release and reactivation in temperate trees are mainly controlled by temperature and are affected by age, but the underlying molecular mechanisms are still unclear. In this study, we explored the effects of low temperatures in winter and warm temperatures in spring on dormancy release and reactivation in *Larix kaempferi*. Further, we established the relationships between cell-cycle genes and cambium cell division. The results showed that chilling accelerated *L. kaempferi* bud break overall, and the longer the duration of chilling is, the shorter the bud break time is. After dormancy release, warm temperatures induced cell-cycle gene expression; when the configuration value of the cell-cycle genes reached 4.97, the cambium cells divided and *L. kaempferi* reactivated. This study helps to predict the impact of climate change on wood production and provides technical support for seedling cultivation in greenhouses.

## 1. Introduction

The cyclical alternation of growth and dormancy in temperate trees is regulated by temperature [1,2,3]. Dormant trees require sufficient chilling to release dormancy [4,5]. However, different tree species have different chilling requirements, which are mainly reflected in their responses to chilling [6,7,8]. Temperatures of 0 °C or lower are more effective for dormancy release in *Ribes nigrum* L. [9] and *L. decidua* [6]. *Betula alleghaniensis* has a higher chilling requirement than *L. laricina*, *Pinus strobus*, *P. resinosa*, *Picea rubens*, and *Thuja occidentalis* [7]. In addition, four weeks of chilling induces dormancy release in poplar [4].

Low temperatures in winter induce dormancy release in trees, and warm temperatures in spring induce their reactivation [10,11,12]. Water culture at 25 °C induces bud break in poplar branches [12], and the longer the chilling time is, the more the buds break [4]. Localized heating of dormant stems of *Cryptomeria japonica* [13], *L. kaempferi* (Japanese larch) [14], and *Quercus serrata* [2] induces cambium cell division of the heated area, and the longer the chilling time is, the faster the cambium cell division occurs [14,15]. These results suggest that tree dormancy release and reactivation are synergistically regulated by winter and spring temperatures [5]. Tree reactivation is the beginning of wood formation [16], and climate change reduces the occurrence of winter low temperatures, so studying the regulation of tree reactivation by temperature can help to predict the effects of climate change on wood production [17].

In addition, tree reactivation is affected by age [13,18,19,20]. Young *Abies georgei* var. *Smithii* (43 years) [18] and *C. Japonica* (55 years) [13] trees reactivate earlier than do old *A. georgei* (162 years) and *C. japonica* (80 years) trees. Adult *L. decidua*, *P. cembra*, and *P. abies* trees (50–80 years) show xylem differentiation 2–3 weeks earlier than do older trees (200–350 years) [20]. These results suggest that age influences the timing of reactivation, likely by affecting tree responses to the environmental temperature [21].

Cell division is the result of the concerted regulation of cell-cycle genes [22]. Cell-cycle genes mainly include cyclins (CYCs) and cell-cycle protein-dependent kinases (CDKs) [5,22]. As a catalytic subunit, CDK needs to bind to specific CYCs to gain activity and activate the next set of CDK-CYC complexes to promote cell-cycle progression [22]. This suggests that cell division requires the synergistic regulation of cell-cycle genes [22,23,24]. *PtoCDKB* and *PtoCYCB* have seasonal expression patterns in poplar, and their expression levels gradually increase with increasing temperatures during tree reactivation [25,26], suggesting that their expression and functions are not only synchronized with the season or the environment but also coordinated with cambium cell division and wood formation [5]. In our previous study, 13 cell-cycle genes were identified in *L. kaempferi*, including 4 *CDKs* and 9 *CYCs* [5]. It has been found that spring temperatures induce the expression of *LaCDKB1;3* and *LaCYCB1;1* after the chilling requirement is satisfied and that there is an interaction between LaCDKB1;3 and LaCYCB1;1 [5]; these data suggest that winter and spring temperatures synergistically regulate the expression of *LaCDKB1;3* and *LaCYCB1;1* and that the acquisition of LaCDKB1;3 activity requires LaCYCB1;1 [5]. The interactions between the other 11 cell-cycle genes and their relationships with temperature are unknown.

In this study, the interactions of 13 cell-cycle genes and their responses to temperature were analyzed to explore the regulatory mechanism of winter and spring temperatures on the dormancy release and reactivation of *L. kaempferi*, to determine the chilling requirement of *L. kaempferi*, and to establish the relationships between cell-cycle gene expression and cambium cell division. In addition, the effect of age on *L. kaempferi* reactivation was analyzed. The results not only help to elucidate the molecular basis of the temperature-synergistic regulation of tree reactivation but also provide technical support for forest stand management and seedling cultivation in greenhouses.

## 2. Results

### 2.1. Low-Temperature Treatment Accelerates Bud Break in Seedlings Overall

**Figure 1:**
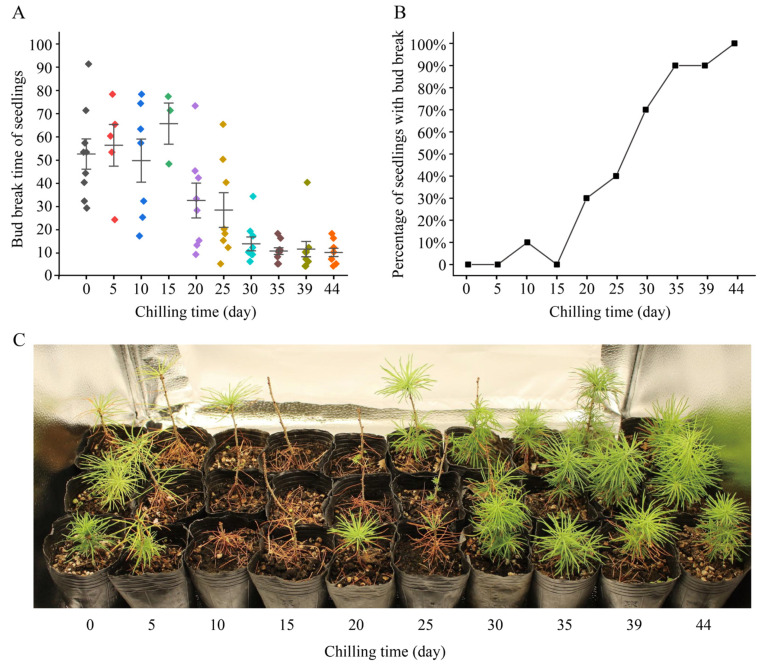
Bud break and growth status of *Larix kaempferi* seedlings treated with low temperature. (**A**) Bud break times of *L. kaempferi* seedlings. Each point represents the bud break time of a seedling, and one color indicates one low-temperature treatment. Error bars represent the SDs. (**B**) Percentage of seedlings with bud break after 18 days of warm-temperature treatment. (**C**) Growth status of *L. kaempferi* seedlings after 93 days of temperature treatment.

A seedling was considered to reactivate when a dormant bud on the seedling expanded, bud scales opened, and green needles were exposed (Figure S1). We observed and recorded the bud break of each seedling in the low-temperature treatment experiments. Up to 3 March 2023, when the experiment ended, a total of 2 seedlings had died, so we counted a total of 98 seedlings. In 22 of the 98 seedlings, bud break did not occur, and 20 of these 22 seedlings received chilling for less than 30 days (Table 1). Seedlings with 0 or 5 days of chilling had not yet lost their needles produced in the previous year; those with 10 or 15 days of chilling had lost most of their needles; in those with 20 or 25 days of chilling, bud break was observed but not yet enough new needles were produced; and those with ≥30 days of chilling had produced enough new branches and needles (Figure 1C).

We found that when the chilling time was <30 days, the bud break time of the seedlings was relatively dispersed, while when the chilling time was >30 days, the bud break time of the seedlings was concentrated (Table 1, Figure 1A). Without chilling, nine seedlings (90%) had bud break, and the bud break time ranged 29 to 91 days (Table 1). With 30 days of chilling, nine seedlings (90%) had bud break, and the bud break time ranged from 6 to 34 days (Table 1). With 44 days of chilling, eight seedlings (100%) had bud break, and the bud break time ranged from 4 to 18 days (Table 1).

Further analysis showed that the time required for *L. kaempferi* bud break decreased gradually with an increase in the chilling time. When the chilling time was 10 days, the average bud break time was 49 days; when the chilling time was 30 days, it was 14 days; and when the chilling time was 44 days, it was 10 days (Table 1; Figure 1A). The correlation analysis showed that the chilling time negatively correlated with the bud break time, with a correlation coefficient of −0.901 (*p* < 0.01). These data indicate that the longer the chilling time is, the shorter the bud break time is.

In seedlings with 44 days of chilling, bud break occurred in each seedling within 18 days (Table 1; Figure 1A). We counted the percentage of seedlings with bud break at 18 days for each treatment. When the chilling time was ≤30 days, the percentage of seedlings with bud break did not exceed 40%; when the chilling time was 30 days, it was 70%; and when the chilling time was 35 or 39 days, it was 90% (Figure 1B). These data indicate that the percentage of seedlings with bud break gradually increased with an increase in chilling time.

### 2.2. Cell-Cycle Genes Are Regulated by Temperature

**Figure 2:**
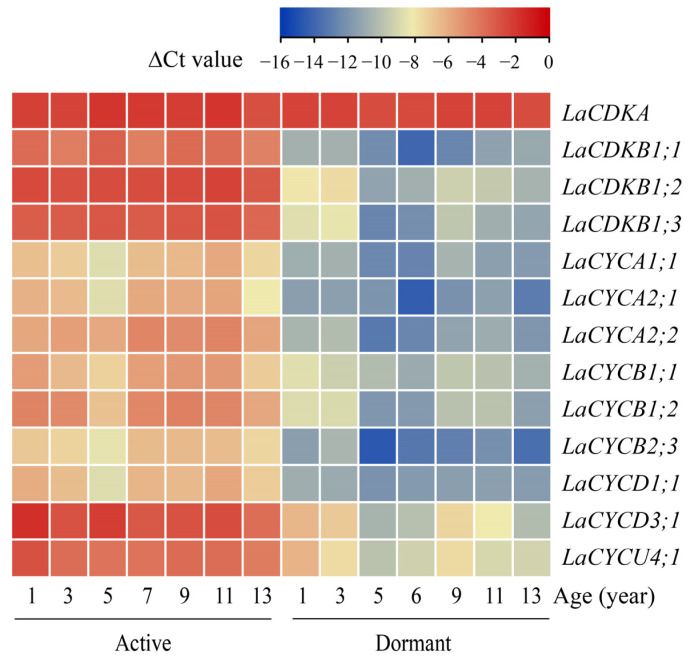
Expression patterns of 13 cell-cycle genes examined by qRT-PCR in active (sampled on 4 July 2019) and dormant (sampled on 11 November 2020) *L. kaempferi* stems, with *L. kaempferi* ubiquitin-conjugating enzyme E2 28 (*LaUBC1*) as the internal reference gene. All the stems were newly produced in the corresponding year.

The quantitative reverse transcription polymerase chain reaction (qRT-PCR) results showed that the expression level of *LaCDKA* did not differ between the active and dormant stages (*p >* 0.05), whereas the expression levels of the other 12 cell-cycle genes were high in the active stage and low in the dormant stage (*p* < 0.05) (Figure 2).

**Figure 3:**
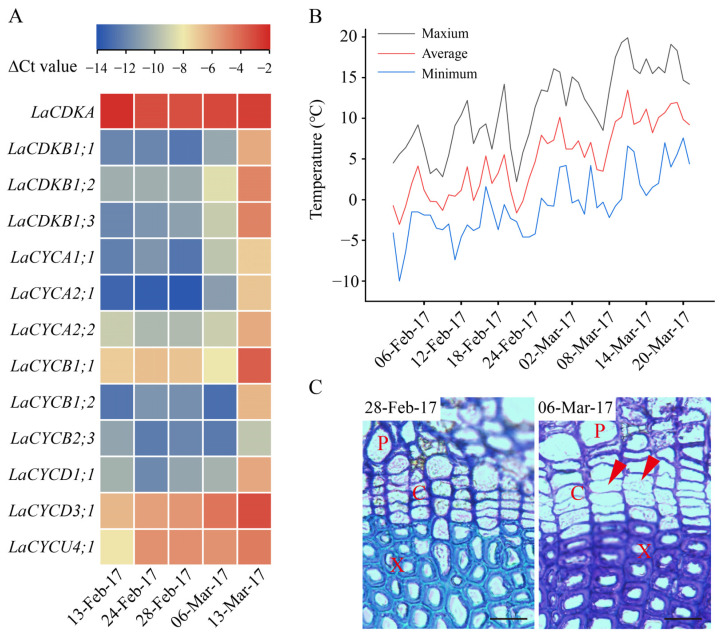
Expression patterns of 13 cell-cycle genes during *L. kaempferi* cambium reactivation from 13 February to 13 March 2017. (**A**) Heatmap showing the expression patterns of 13 cell-cycle genes assayed by qRT-PCR. (**B**) Climatic daily temperatures from 13 February to 13 March 2017 [5]. (**C**) Photomicrographs of cambium regions in a cross-section in the dormant stage (28 February 2017) and reactivation stage (6 March 2017) of *L. kaempferi*. Red arrowheads indicate thin, newly formed cell walls. P, phloem; X, xylem; C, cambium. Scale bar, 20 µm.

From 13 February to 13 March 2017, the expression levels of *LaCDKA* and *LaCYCB2;3* did not increase (*p >* 0.05), while those of the other 11 genes increased (*p* < 0.05) (Figure 3A). The expression levels of *LaCYCD3;1* and *LaCYCU4;1* increased on 24 February 2017 (*p* < 0.05); that of *LaCDKB1;3* increased on 28 February 2017 (*p* < 0.05); those of *LaCDKB1;1*, *LaCDKB1;2*, *LaCYCA1;1*, and *LaCYCA2;1* increased on 6 March 2017 (*p* < 0.05); and those of *LaCYCA2;2*, *LaCYCB1;1*, *LaCYCB1;2*, and *LaCYCD1;1* increased on 13 March 2017 (*p* < 0.05) (Figure 3A).

**Figure 4:**
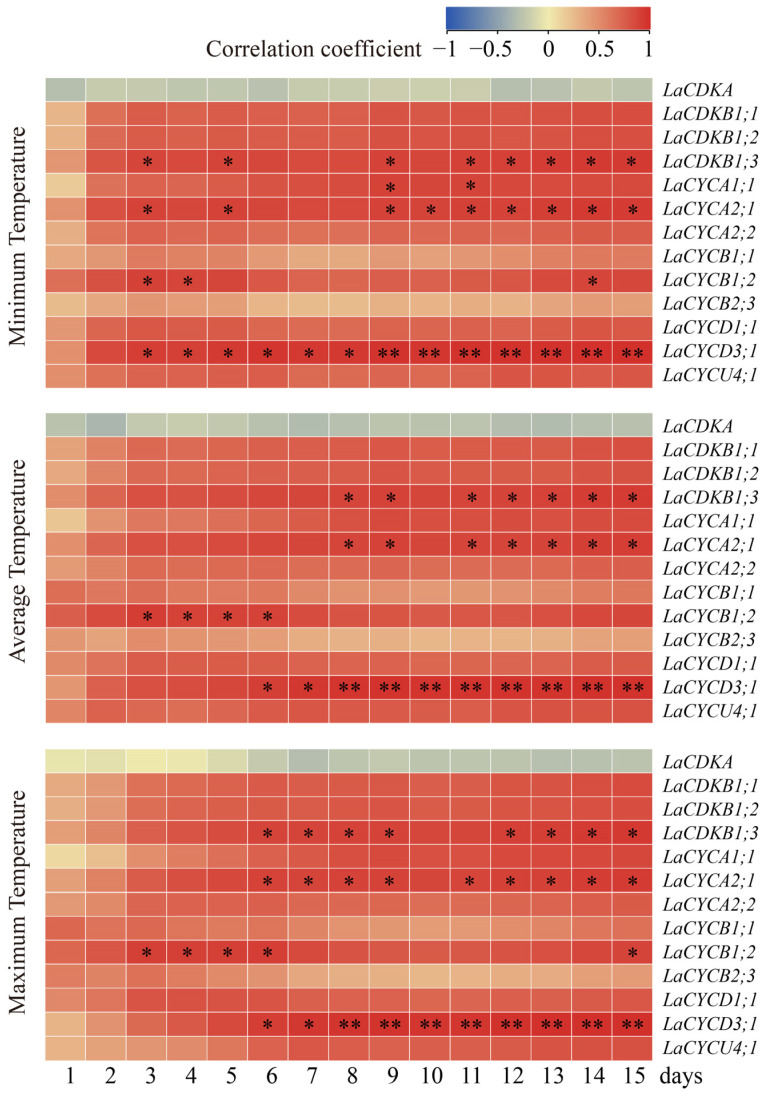
Correlations between the expression levels of 13 cell-cycle genes and the minimum temperature, average temperature, and maximum temperature from 13 February to 13 March 2017. * and ** mean that the correlations between gene expression and temperature were significant at *p* < 0.05 and *p* < 0.01, respectively.

To assess the relationships between temperatures and cell-cycle gene expression, we carried out a Pearson correlation analysis. The results showed that the expression levels of *LaCDKB1;3*, *LaCYCA1;1*, *LaCYCA2;1*, *LaCYCB1;2*, and *LaCYCD3;1* positively correlated with the temperature (*p* < 0.05) (Figure 4). The expression level of *LaCDKB1;3* correlated with the average minimum temperature for at least 3 days (*p* < 0.05); that of *LaCYCA1;1* correlated with the average minimum temperature for at least 9 days (*p* < 0.05); that of *LaCYCA2;1* correlated with the average minimum temperature for at least 3 days (*p* < 0.05); that of *LaCYCB1;2* correlated with the average minimum, average, and maximum temperatures for at least 3 days (*p* < 0.05); and that of *LaCYCD3;1* correlated with the average minimum temperature for at least 3 days (*p* < 0.05) and correlated with the average maximum temperature for at least 8 days (*p* < 0.01) (Figure 4).

Before 28 February 2017, the cell walls in the cambium regions were thick and no dividing cambium cells were observed, whereas on 6 and 13 March 2017, cambium cells with thinner cell walls were observed (Figure 3C).

### 2.3. Water Culture Induces Cambium Reactivation and Cell-Cycle Gene Expression in L. kaempferi

**Figure 5:**
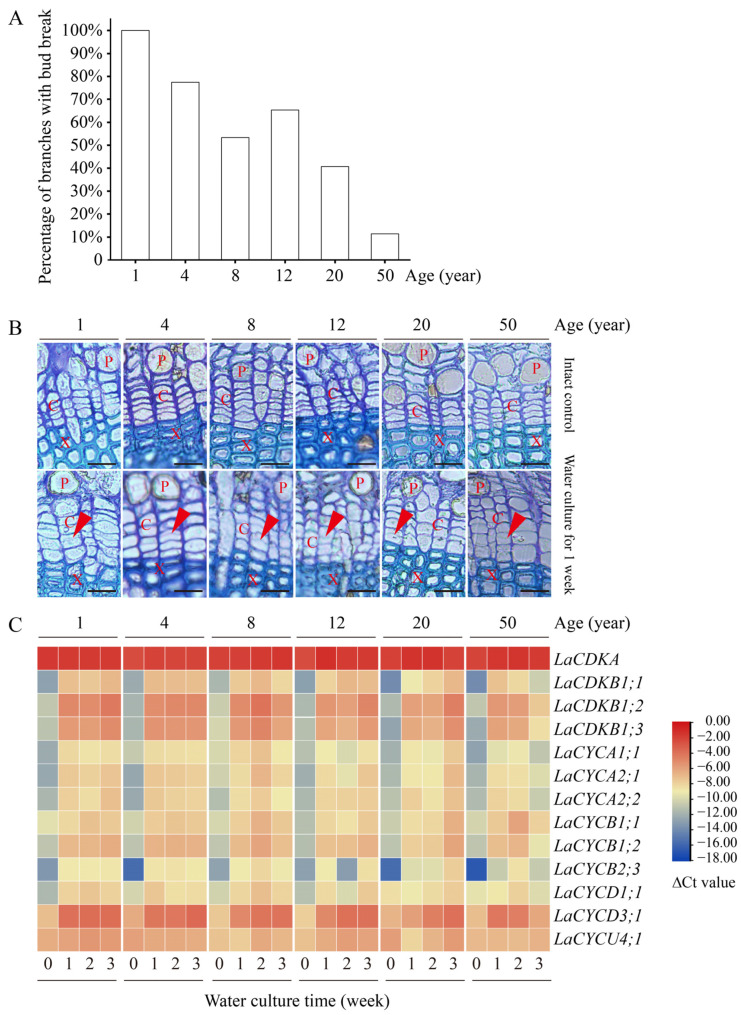
Bud and cambium reactivation and the expression patterns of 13 cell-cycle genes in the branches from 1-, 4-, 8-, 12-, 20-, and 50-year-old dormant *L. kaempferi* trees after water culture. (**A**) Percentage of *L. kaempferi* branches with bud break after water culture for 9 days. (**B**) Photomicrographs of cambium regions in cross-sections showing cambium reactivation after water culture for one week. (**C**) Variation in the cell-cycle gene expression levels in *L. kaempferi* stems after water culture. For the water culture experiment, branches from 1-, 4-, 8-, 12-, 20-, and 50-year-old dormant trees were harvested on 10 March 2013 and then taken to the laboratory for water culture [27]. Red arrowheads indicate thin, newly formed cell walls. P, phloem; X, xylem; C, cambium. Scale bar, 20 µm.

Water culture can induce bud break in *L. kaempferi*. After 9 days of water culture, all branches from 1-year-old trees had bud break, while only 11% of branches from 50-year-old trees had bud break (Figure 5A). A correlation analysis showed that the percentage of branches with bud break negatively correlated with tree age (*p* < 0.05), with a correlation coefficient of −0.91.

Water culture can induce cambium cell division. Before the water culture, the walls of the cambium cells were thick and no dividing cells were observed, while after one week of water culture, dividing cells were observed in all branches (Figure 5B).

Water culture can induce cell-cycle gene expression. The expression levels of all the examined cell-cycle genes, except for *LaCDKA* and *LaCYCU4;1*, increased after one week of water culture (*p* < 0.05) (Figure 5C).

### 2.4. Cambium Reactivation Is Closely Related to the Cell-Cycle Gene Configuration

To elucidate the synergistic effect of cell-cycle genes on cambium cell division, we analyzed the relationships between the expression patterns of 12 seasonally expressed cell-cycle genes and cell division. By comparing the ∆CT values of the 12 genes in the qRT-PCR results, the minimum and maximum ∆CT values of each gene were determined. Then, their expression levels were normalized based on the following Formula (1):

X_gene_ = (∆CT value − minimum ∆CT value)/(maximum ∆CT value − minimum ∆CT value) (1)

After normalization, the expression level of each gene (X_gene_) in one sample ranged from 0 to 1. Thus, the configuration value of 12 cell-cycle genes in one sample was obtained based on the following Formula (2):

Configuration value = x_*LaCDKB*1*;*1_ + x_*LaCDKB*1*;*2_ + x_*LaCDKB*1*;*3_ + x_*LaCYCA*1*;*1_ + x_*LaCYCA*2*;*1_ + x_*LaCYCA*2*;*2_ + x_*LaCYCB*1*;*1_ + x_*LaCYCB*1*;*2_ + x_*LaCYCB*2*;*3_ + x_*LaCYCD*1*;*1_ + x_*LaCYCD*3*;*1_ + x_*LaCYCU*4*;*1_ (2)

**Figure 6:**
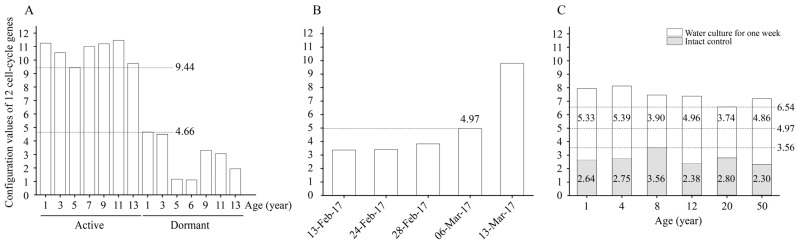
The configuration values of 12 cell-cycle genes in *L. kaempferi*. (**A**) The configuration values of 12 cell-cycle genes in the active and dormant stages of *L. kaempferi*. (**B**) The configuration values of 12 cell-cycle genes during *L. kaempferi* seedling reactivation from 13 February to 13 March 2017. (**C**) The configuration values of 12 cell-cycle genes during water culture of *L. kaempferi* branches harvested from 1-, 4-, 8-, 12-, 20-, and 50-year-old dormant trees on 10 March 2013 [27]. 9.44 was the lowest configuration value in the active stage, 4.66 was the highest in the dormant stage; when it was 4.97 on 6 March 2017, cambium cell division was detected; 3.56 was the highest on 13 March 2013, and 6.54 was the lowest after one week of water culture.

By calculating the configuration values of the 12 cell-cycle genes in the active and dormant stages of *L. kaempferi* aged from 1 to 13 years, we found that the configuration values were >9.44 in the active stage and <4.66 in the dormant stage (Figure 6A).

From 13 February to 13 March 2017, the configuration value of the 12 cell-cycle genes gradually increased from 3.37 to 9.80; on 6 March 2017, when the cambium cells had already divided, it was 4.97 (Figure 6B).

When the water culture experiments were started on 13 March 2013, the highest configuration value of the 12 cell-cycle genes was 3.56; after one week of water culture, the configuration values in all materials increased, with a minimum of 6.54 (Figure 6C).

**Figure 7:**
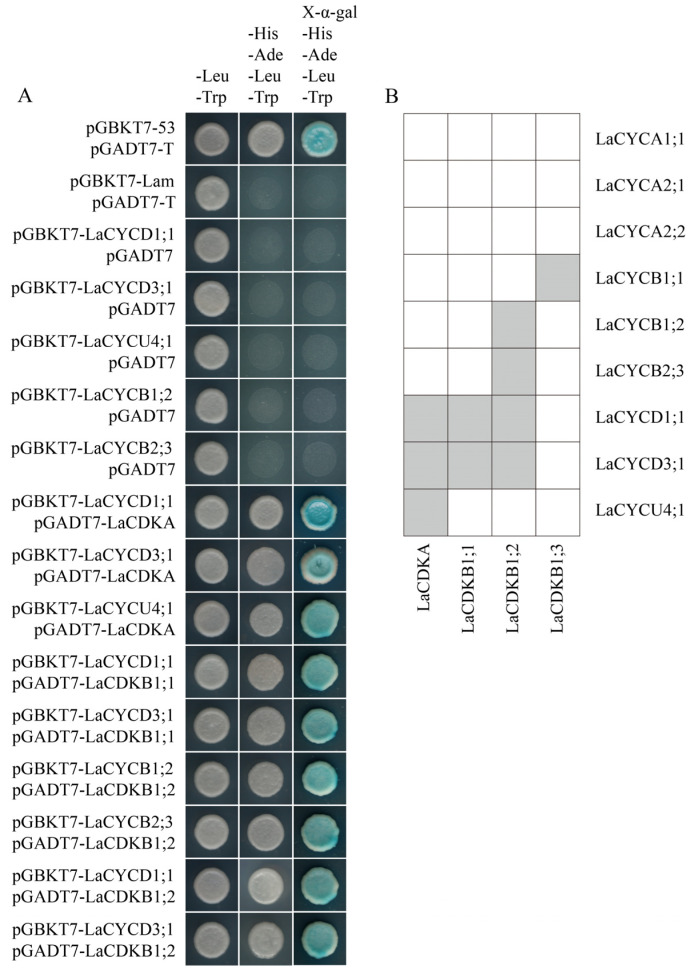
Analysis of protein interaction. (**A**) Yeast two-hybrid assays showed that LaCDKA interacted with LaCYCD1;1, LaCYCD3;1, and LaCYCU4;1; LaCDKB1;1 interacted with LaCYCD1;1 and LaCYCD3;1; and LaCDKB1;2 interacted with LaCYCB1;2, LaCYCB2;3, LaCYCD1;1, and LaCYCD3;1. (**B**) Summary of the analysis results of protein interactions between LaCDKs and LaCYCs [5].

The results of yeast two-hybrid assays and bimolecular fluorescence complementation assays showed that LaCDKA interacted with LaCYCD1;1, LaCYCD3;1, and LaCYCU4;1; LaCDKB1;1 interacted with LaCYCD1;1 and LaCYCD3;1; and LaCDKB1;2 interacted with LaCYCB1;2, LaCYCB2;3, LaCYCD1;1, and LaCYCD3;1 (Figure 7A; Figure S2). In addition, interactions of LaCDKB1;3 with LaCYCB1;1 have been reported [5] (Figure 7B).

## 3. Discussion

The dormancy release and reactivation of *L. kaempferi* are regulated by temperature [5], but the underlying molecular mechanisms are still unclear. In this study, we not only explored the regulatory effects of temperature on the dormancy release and reactivation of *L. kaempferi* but also established the relationships between the cell-cycle gene configuration and cell division. These results provide evidence for elucidating the molecular mechanisms of the temperature-induced dormancy release and reactivation of trees.

### 3.1. Chilling Induces the Transition of L. kaempferi from the Rest to the Quiescence Stage

Baumgarten proposed that the main factor determining the dormancy release in trees is the duration of low temperature, rather than the actual temperature experienced [6]. Therefore, we investigated the effect of the duration of low temperature on dormancy release in *L. kaempferi.* The results showed that the longer the duration of low temperature was, the shorter the bud break time was, which is consistent with the results from other temperate tree species [7,17,28,29,30,31] (Table 1; Figure 1A). Our data show that a low temperature in winter is very important for spring bud break in *L. kaempferi*, and different durations of low temperature induce dormancy release to different extents.

Tree dormancy consists of two main stages: rest and quiescence [4,25,32]. The effect of low temperature is to induce the transition from rest to quiescence, resulting in the loss of tree insensitivity to growth-promoting factors [4,32,33]. Without chilling, it would take a long time for trees to release dormancy [34], which may be related to the regulation of cell-cycle genes [5,25,35], auxin-binding protein 1 [36], and plasmodesmata [33,37,38] by low temperature.

In this study, when the chilling time was <30 days, the bud break time of *L. kaempferi* was longer, and the average bud break time was >28 days; when the chilling time was >30 days, the bud break time was shorter, and the average bud break time was <11 days (Table 1; Figure 1A). These results indicate that chilling for 30 days can satisfy the chilling requirement of *L. kaempferi* and cause *L. kaempferi* to enter into the quiescence stage from the rest stage and gain sensitivity to growth-promoting factors. These findings provide theoretical and technological support for *L. kaempferi* cultivation in greenhouses.

### 3.2. Spring Temperatures Induce Cell-Cycle Gene Expression in L. kaempferi

From 13 February to 13 March 2017, the expression levels of all the examined cell-cycle genes, except for *LaCDKA* and *LaCYCB2;3*, increased (*p* < 0.05), but the timing of the increases differed (Figure 3A). These data indicate that the expression of cell-cycle genes is induced by temperature during *L. kaempferi* reactivation, but these genes have different response patterns to temperature [5].

The correlation analysis showed that the expression levels of *LaCDKB1;3*, *LaCYCA1;1*, *LaCYCA2;1*, *LaCYCB1;2*, and *LaCYCD3;1* positively correlated with temperature (Figure 4). Despite having different correlation patterns, their expression levels all correlated with the average minimum temperature for at least 3 days (*p* < 0.05) (Figure 4), suggesting that cell-cycle genes are regulated by cumulative rather than transient temperatures [5]. The differential expression of the 12 cell-cycle genes between the active and dormant stages reflects their regulation by environmental signals and demonstrates their important roles in seasonal growth in trees, especially in the regulation of cambium activity [5] (Figure 2).

### 3.3. Cell-Cycle Genes Cooperatively Regulate Cell Division

Correlation analysis was applied to the cell-cycle gene expression levels and cambium cell division (0 for no division, 1 for division) during *L. kaempferi* reactivation in the spring of 2017; the normalized expression levels of all 12 cell-cycle genes positively correlated with cambium cell division (*p* < 0.01), indicating that these genes play roles in regulating cambium cell division. Therefore, it is reliable to use the configuration value of the 12 cell-cycle genes to show their overall expression levels, and it is feasible to explore the relationships between these genes’ configuration and cambium cell division.

During the active stage, the cambium cells divide vigorously [5], and the configuration values of the 12 cell-cycle genes were all >9.44; whereas during the dormant stage, the cambium cells stop dividing [5], and the configuration values of these genes were all <4.66 (Figure 6A). We speculate that there is a configuration value between 4.66 and 9.44 such that when this value is reached, the cambium cells start to divide.

From 13 February to 13 March 2017, the configuration values of the 12 cell-cycle genes gradually increased, and cambium cell division occurred on 6 March 2017, at which time the value of these genes was 4.97 (Figure 6B). Therefore, we suggest 4.97 as the threshold value; that is to say, the cambium cells start to divide when the configuration value of the 12 cell-cycle genes reaches 4.97.

When the water culture experiments were started on 13 March 2013, the cambium cells did not divide, and all the configuration values of the 12 cell-cycle genes were <4.97; after one week of water culture, the cambium cells had divided in all branches, and the values of these genes were all >4.97 (Figure 6C). These results further confirm our speculation.

The cell cycle has four phases: G1, S, G2, and M [22]. The transitions from G1 to S and from G2 to M are key checkpoints in the cell-cycle progression and are regulated by cell-cycle genes [5,22,39,40]. Previous studies have shown that CDKA-CYCD complexes function in the G1-to-S transition and CDKA/B-CYCB complexes function in the G2-to-M transition [22,40]. During *L. kaempferi* reactivation, the expression level of *LaCDKA* did not increase (*p >* 0.05), whereas the expression levels of its interacting genes *LaCYCD1;1*, *LaCYCD3;1*, and *LaCYCU4;1* increased (*p* < 0.05). Notably, the timings of their up-regulation were different. *LaCYCD3;1* and *LaCYCU4;1* were up-regulated on 24 February (*p* < 0.05), and *LaCYCD1;1* was up-regulated on 13 March (Figure 3A). The up-regulation timings of *LaCDKB1;1*, *LaCDKB1;2*, *LaCDKB1;3*, and their interacting genes were also different (Figure 3A). These results not only showed the processes of cell-cycle progression during tree reactivation but also indicated the complexity and flexibility of the temperature control of tree reactivation by regulating cell-cycle genes [5].

The expression levels of *LaCYCD3;1* and *LaCYCU4;1* increased earlier than did those of the cell-cycle genes (*CDKB* and *CYCB*) involved in the G2-to-M transition (Figure 3A), indicating that the G1-to-S transition occurs earlier than the G2-to-M transition, and that LaCDKA-LaCYCU4;1 complexes might also function in the G1-to-S transition.

There was no correlation between the *LaCYCU4;1* expression level and temperature (*p >* 0.05), which might result from the earlier increase in the *LaCYCU4;1* expression level on 24 February. When the water culture experiments were started on 13 March 2013, the normalized expression level of *LaCYCU4;1* in 4- and 20-year-old *L. kaempferi* exceeded 0.56. These materials were collected on 10 March 2013 in northeast China and then taken to Beijing. We deduced that the expression level of *LaCYCU4;1* had increased before 10 March 2013 or during the transport of the materials.

### 3.4. Age Affects the Timing of Bud Reactivation and Orchestrates the Configuration Process of Cell-Cycle Genes

In the water culture experiment, the bud break time negatively correlated with age (*p* < 0.01), suggesting that the timing of *L. kaempferi* bud reactivation is affected by age. It has been reported that age also affects the timing of cambium cell division and xylem cell differentiation [18,19,20,21,41]. However, in this water culture experiment, we did not find a difference in the timing of cambium cell division in *L. kaempferi* branches sampled from trees of different ages. This might result from the late sampling, and one week is too long to capture the difference in the timing of cambium reactivation.

Notably, differences in the changes in the cell-cycle gene configuration were captured. After one week of water culture, the configuration values of the 12 cell-cycle genes in branches of 1- and 4-year-old *L. kaempferi* increased by 5.33 and 5.39, respectively, showing greater increases than those in branches of 8-, 12-, 20-, and 50-year-old *L. kaempferi* (Figure 6C). These data suggest that the configuration process of cell-cycle genes is influenced by age, which might be one of the molecular bases of the influence of age on tree reactivation. Based on these data, here, we conclude that the cell-cycle gene configuration value increases faster in young trees than in old trees; therefore, it reaches the threshold value earlier in young trees, resulting in earlier reactivation.

## 4. Materials and Methods

### 4.1. Sample Treatment and Collection

#### 4.1.1. Natural Chilling Treatment Experiments

To explore the chilling requirement of *L. kaempferi* in winter, we conducted a low-temperature treatment experiment using *L. kaempferi* seedlings in Beijing (39°48′ N, 116°28′ E). On 30 November 2022, 10 seedlings were moved from the greenhouse to a growth chamber, and 90 seedlings were moved outdoors. These 100 seedlings were grown from seeds in April 2022 in the greenhouse and not exposed to temperatures below 13 °C. After 5, 10, 15, 20, 25, 30, 35, 39, or 44 days of natural chilling treatment, 10 seedlings were moved into the growth chamber and watered daily as needed. The average daily temperature in the natural chilling treatment experiments did not exceed 4 °C (Figure S3). In the growth chamber, a growth system was set up with a 16 h photoperiod, temperatures of 25/20 °C (day/night), and relative humidity of 75%. From 30 November 2022 to 3 March 2023, the number of new bud breaks of each seedling was counted daily. We used Statistical Product and Service Solutions (SPSS Statistics 26, IBM Corp. Armonk, NY, USA) to apply Pearson correlation analysis to the bud break time and the chilling time.

#### 4.1.2. Materials Collected from the Active and Dormant *L. kaempferi* Trees

To study the expression patterns of cell-cycle genes in the active (sampled on 4 July 2019) and dormant (sampled on 11 November 2020) *L. kaempferi* trees, we used the materials which were collected in our previous studies [27,42]. These trees were grown from seeds and located in Dagujia seed orchard (42°22′ N, 124°51′ E), Liaoning Province, in Northeast China. When sampling, the buds or needles were removed, and the stems were cut into pieces, frozen in liquid nitrogen, and stored at −80 °C until RNA extraction.

The expression levels of cell-cycle genes in the active and dormant materials were assessed by qRT-PCR, and then they were analyzed using Student’s *t*-test.

#### 4.1.3. Materials Collected during Natural Reactivation of *L. kaempferi* Trees

To study the effects of spring temperatures on cell-cycle gene expression and vascular cambium activity, we also used the materials that were collected in our previous study [5]. One-year-old pot-grown dormant seedlings 19 cm in mean length, which were grown in Beijing, were sampled from 13 February to 20 March in 2017 at six time points. Five seedlings were sampled each time. The sampling method was the same as above. At the same time, small blocks of vascular tissue were excised from the middle of dormant branches and fixed in formalin–alcohol–acetic acid for anatomical observations. In this study, materials sampled from five time points were used because the materials sampled on 20 March 2017 were used up.

#### 4.1.4. Water Culture Experiments

To investigate the effects of age on *L. kaempferi* reactivation and cell-cycle gene expression, we conducted water culture experiments using 1-, 4-, 8-, 12-, 20-, and 50-year-old dormant *L. kaempferi* trees. Parts of these experiments were described in our previous study [27], and here we gave a detailed description again. Branches from dormant *L. kaempferi* trees were harvested on 10 March 2013 in Dagujia seed orchard and then taken to Beijing. At least 8 branches from each age category were sampled as the intact control on 13 March 2013 after the removal of all buds. On 13 March 2013, water culture experiments were performed, and at least 7 branches from each age category were cultured with or without buds. Branches without buds were treated with lanolin, which was spread over the excised tops of the branches. A water culture system was set up in a growth chamber with a 16 h photoperiod, temperatures of 25/20 °C (day/night), and a relative humidity of 75%. Branches were sampled after culture for 1, 2, or 3 weeks as above for RNA extraction and anatomical observations. Bud break in the branches with buds was counted daily. We used SPSS 26 to apply Pearson correlation analysis to the percentage of branches with bud break within nine days and the trees’ age. The expression levels of cell-cycle genes in each sample were assessed by qRT-PCR, and then they were analyzed using a one-way ANOVA and Duncan’s test.

### 4.2. RNA Extraction and cDNA Synthesis

After the materials were ground in liquid nitrogen, the RNA was extracted using the EasyPure RNA Kit (TransGen Biotech, Beijing, China) according to the manufacturer’s protocol. A quantity of 2 μg of RNA was reverse-transcribed into cDNA with the TransScript II One-step gDNA Removal and cDNA Synthesis SuperMix Kit (TransGen Biotech, Beijing, China) and subsequently diluted for qRT-PCR.

### 4.3. qRT-PCR

The qRT-PCR experiment was performed with *L. kaempferi* ubiquitin-conjugating enzyme E2 28 as the internal reference gene [27]. A Bio-Rad CFX96 PCR system was used with TB Green^®^ Premix Ex Taq™ (Tli RNase H Plus) (Takara, Shiga, Japan). Each reaction was carried out on 2 µL of diluted cDNA sample, in a total reaction system of 25 µL. The reaction procedure was set up according to the manufacturer’s protocol: 95 °C for 30 s, then 40–45 cycles at 95 °C for 5 s and at 60 °C for 30 s, followed by a melting step from 65 to 95 °C. Four technical replicates were used for each sample, and the ∆CT value (CT_reference gene_ − CT_cell-cycle gene_) was used to show the result. We used TBtools [43] to show the ∆CT value of each gene with a heat map, and different colors correspond to different ∆CT values. The qRT-PCR primers are listed in Table S1.

### 4.4. Anatomical Observations of Secondary Vascular Tissue

The small blocks fixed in formalin–alcohol–acetic acid were dehydrated in an alcohol series and embedded in Spurr’s resin (SPI, West Chester, PA, USA). Cross-sections 4 μm thick were cut on a microtome (Leitz 1512, Wetzlar, Germany), stained with 1% toluidine blue O for 3–4 min, and observed under an Axioskop 2 Plus microscope (Zeiss, Gottingen, Germany) equipped with a computer-assisted digital camera.

### 4.5. Yeast Two-Hybrid Assay

Yeast two-hybrid (Y2H) assays were used to assess the interactions among the cell-cycle genes. The coding sequences of cyclins were cloned into the pGBKT7 vector, and the coding sequences of CDKs were cloned into the pGADT7 vector to fuse with the binding and activation domains, respectively. Then, the bait and prey constructs were co-transformed into yeast strain AH109 using the lithium acetate method, and yeast cells were grown on SD/–Leu–Trp medium for 3–5 days. The positive clones were selected and plated onto SD/−Ade/−His/−Leu/−Trp medium and cultured for 3–5 days, and then positive clones were transferred onto SD/−Ade/−His/−Leu/−Trp medium containing 4 mg mL^−1^ X-α-Gal to test for possible interactions based on the growth status and blue color development in yeast colonies.

### 4.6. Bimolecular Fluorescence Complementation Assay

Bimolecular fluorescent complementation assays were used to directly visualize protein–protein interactions in vivo. The coding sequences of cyclins and CDKs were cloned into the pSM vector to produce the nYFP-cyclin and CDK-cYFP constructs, respectively. Each construct was individually transformed into *Agrobacterium tumefaciens* strain GV3101. Then, the mixed *Agrobacterium* strain was introduced into *Nicotiana benthamiana* leaves via agro-infiltration. After 2 days of incubation, YFP fluorescence was observed in transformed leaf epidermal cells under a laser confocal microscope (Nikon C2-ER). For the proteins for which no fluorescence was observed, their coding sequences were recloned into pSM vectors to produce nYFP-CDK and cyclin-cYFP constructs, respectively, and the experiment was performed again.

### 4.7. Relationship between Cell-Cycle Gene Expression and Temperature

The data for the average, maximum, and minimum daily air temperatures near the experimental site in Beijing, China, were obtained from the National Meteorological Information Center, China Meteorological Administration. Pearson correlation analysis was used to analyze the correlation between the temperature and gene expression with SPSS 26. A *p*-value of <0.05 was considered to indicate a correlation.

## 5. Conclusions

This study determined the regulatory roles of winter and spring temperatures on dormancy release and reactivation in *L. kaempferi* and established the relationship between cell-cycle gene expression and cambium cell division. Winter and spring temperatures coordinately regulate reactivation in *L. kaempferi*. After fulfillment of the chilling requirement in winter, cell-cycle gene expression is induced with the increase in spring temperatures, and when the cell-cycle gene configuration value reaches 4.97, the cambium cells start to divide and *L. kaempferi* reactivates. Age affects the timing of tree reactivation by influencing the cell-cycle gene configuration process. These data reveal the molecular basis of *L. kaempferi* reactivation and provide technical support for seedling cultivation in greenhouses.

## Figures and Tables

**Table 1:** Bud break statistics of *L. kaempferi* seedlings in the low-temperature treatment experiment.

Treatment Time (Days)		Number of Seedlings	Number of Seedlings with Bud Break	Number of Seedlings with No Bud Break	Range of Bud Break Time (Day)	Average Bud Break Time (Day)	Standard Deviation of Bud Break Time
Low Temperature	Warm Temperature						
0	93	10	9	1	29–91	52	19.5
5	88	10	5	5	24–78	56	20.1
10	83	10	7	3	17–78	49	24.5
15	78	10	3	7	48–77	65	15.3
20	73	10	8	2	9–73	32	21.2
25	68	10	8	2	5–65	28	21.1
30	63	10	9	1	6–34	14	8.9
35	58	10	9	1	5–18	10	4.4
39	54	10	10	0	4–40	11	10.5
44	49	8	8	0	4–18	10	5.1

## Data Availability

The sequence data in this study are openly available in NCBI at https://www.ncbi.nlm.nih.gov/, and reference numbers are listed in Table S1.

## Supplementary Materials

The following supporting information can be downloaded at: https://www.mdpi.com/article/10.3390/ijms25073578/s1.
