# Supplementary material for: Cambium Reactivation Is Closely Related to the Cell-Cycle Gene Configuration in Larix kaempferi
Source: Int J Mol Sci. 2024 Mar 22;25(7):3578. doi: 10.3390/ijms25073578 (PMC11011626; doi:10.3390/ijms25073578)
Supplement: Supplementary file 1 [file ijms-25-03578-s001.zip › Legend of Supplementary Figures.pdf]

Figure S1. The criteria for *L. kaempferi* bud break. When the dormant bud reaches the state pointed by the red arrow, bud break is recorded. Scale bar, 1 cm.

Figure S2. Bimolecular fluorescence complementation assays to measure the interactions among the cell-cycle genes.

Figure S3. Climatic daily temperatures from 30 November 2022 to 14 January 2023.

Table S1. The primers of cell-cycle genes.
