# Supplementary figures and images for: Cambium Reactivation Is Closely Related to the Cell-Cycle Gene Configuration in Larix kaempferi
Source: Int J Mol Sci. 2024 Mar 22;25(7):3578. doi: 10.3390/ijms25073578 (PMC11011626; doi:10.3390/ijms25073578)

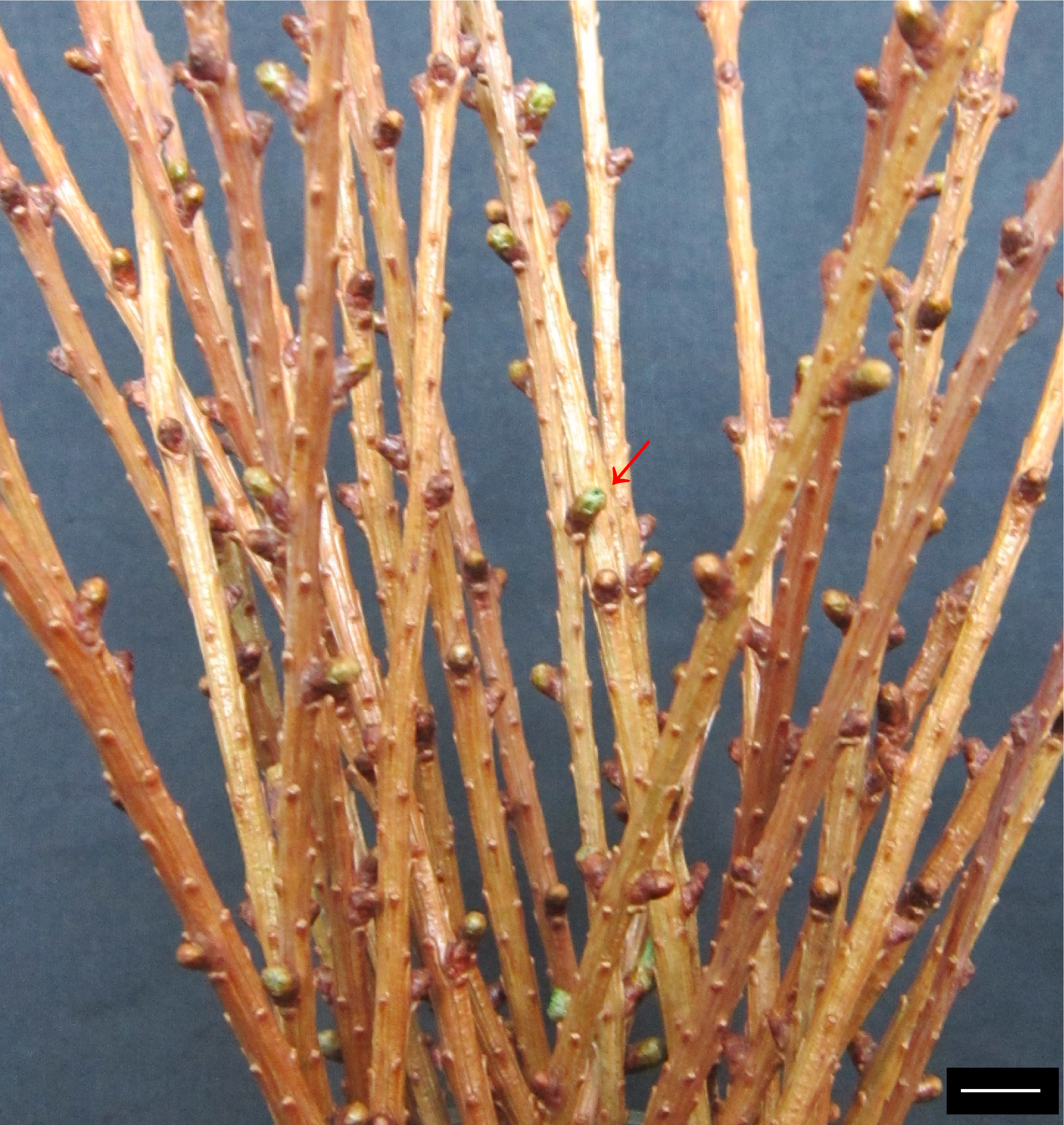

Supplement: Supplementary file 1 [file ijms-25-03578-s001.zip › Figure S1.png]

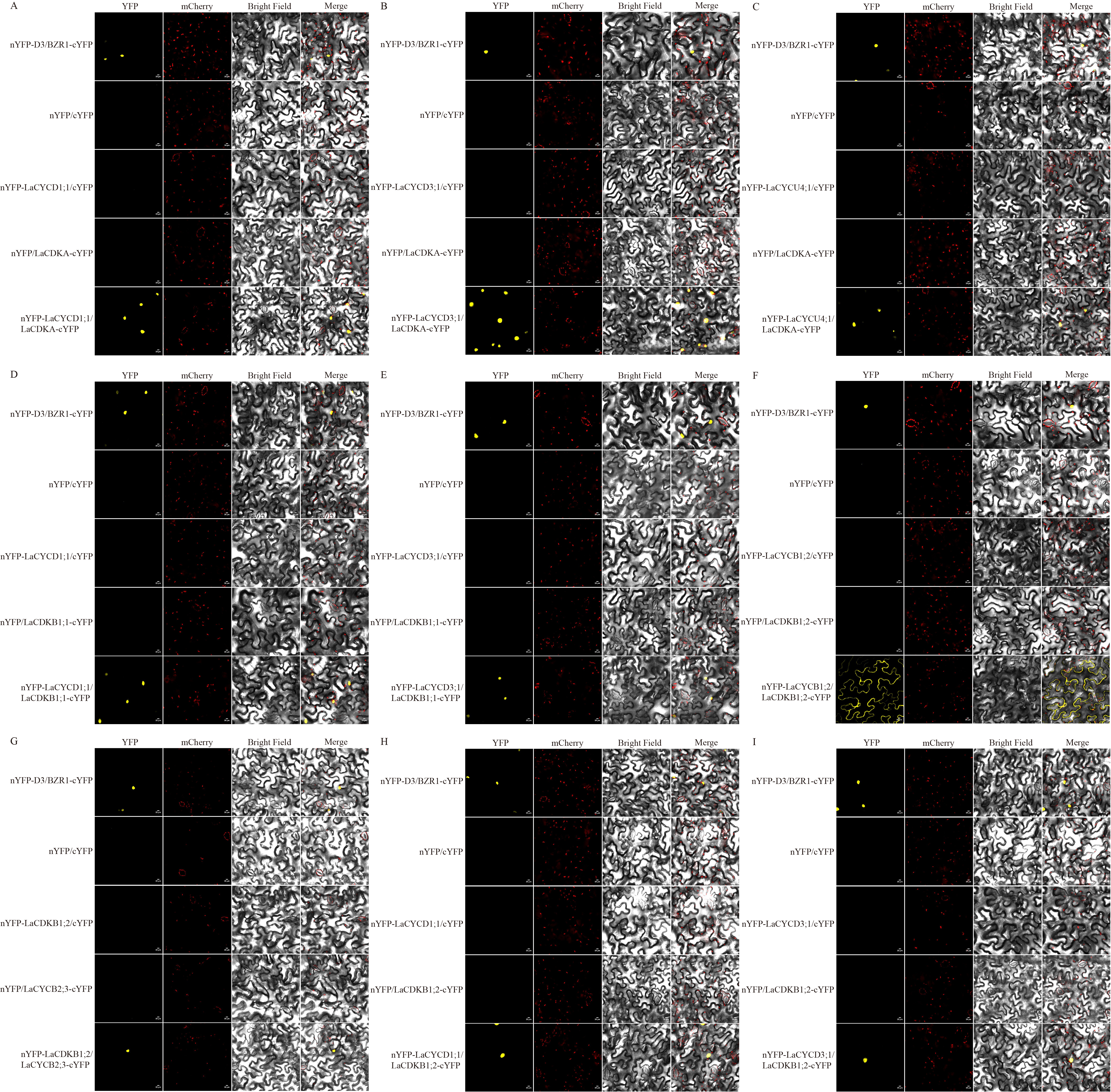

Supplement: Supplementary file 1 [file ijms-25-03578-s001.zip › Figure S2.png]

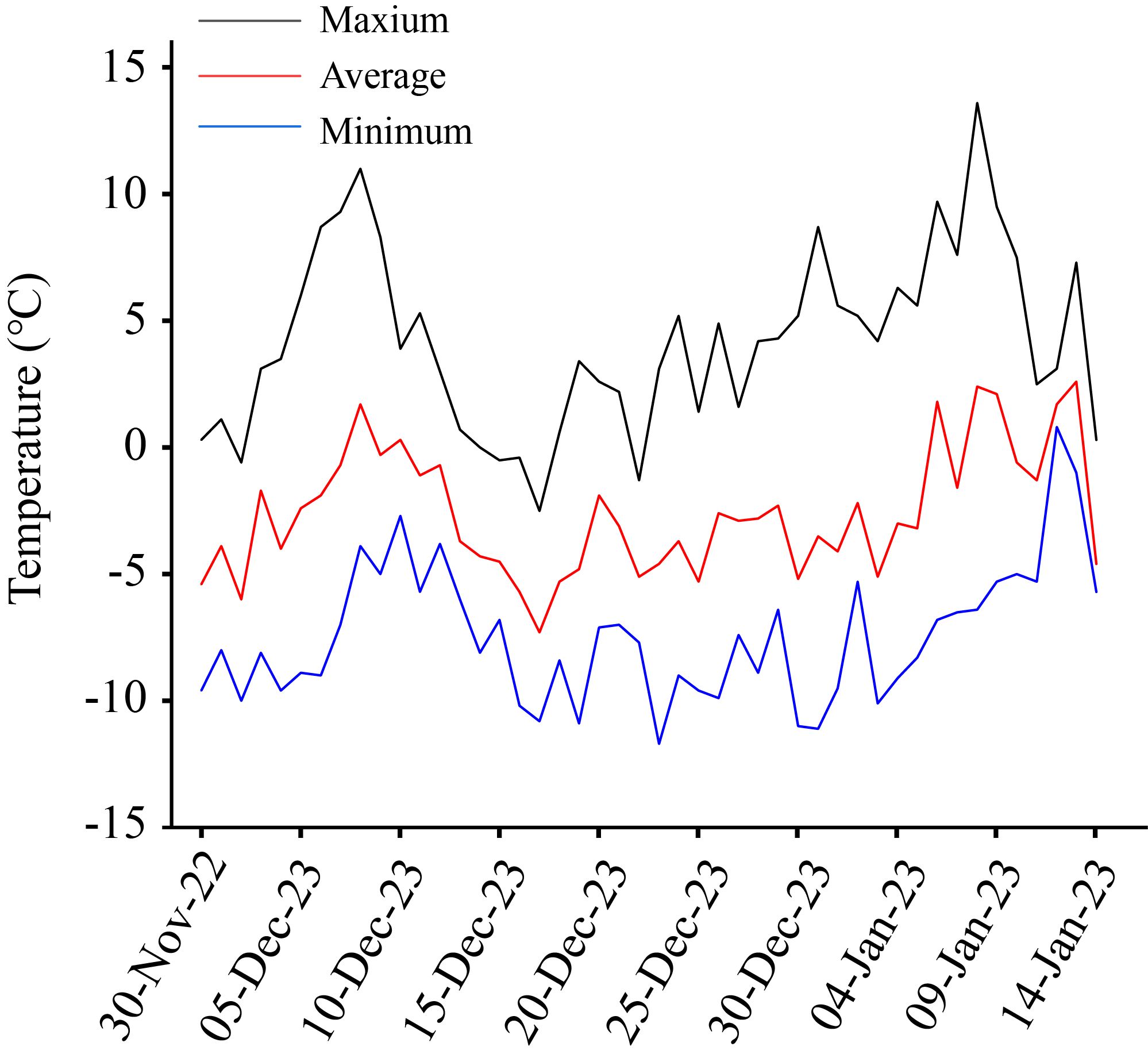

Supplement: Supplementary file 1 [file ijms-25-03578-s001.zip › Figure S3.png]
